# Characterization of Novel OmpA-Like Protein of *Leptospira interrogans* That Binds Extracellular Matrix Molecules and Plasminogen

**DOI:** 10.1371/journal.pone.0021962

**Published:** 2011-07-06

**Authors:** Rosane Oliveira, Zenaide Maria de Morais, Amane Paldes Gonçales, Eliete Caló Romero, Silvio Arruda Vasconcellos, Ana L. T. O. Nascimento

**Affiliations:** 1 Centro de Biotecnologia, Instituto Butantan, São Paulo, Brazil; 2 Interunidades em Biotecnologia, Instituto de Ciências Biomédicas, Universidade de São Paulo, São Paulo, Brazil; 3 Laboratório de Zoonoses Bacterianas do VPS, Faculdade de Medicina Veterinária e Zootecnia, Universidade de São Paulo, São Paulo, Brazil; 4 Centro de Bacteriologia, Instituto Adolfo Lutz, São Paulo, Brazil; Federal University of Minas Gerais, Brazil

## Abstract

*Leptospira interrogans* is the etiological agent of leptospirosis, a zoonotic disease of human and veterinary concern. The identification of novel proteins that mediate host-pathogen interactions is important for understanding the bacterial pathogenesis as well as to identify protective antigens that would help fight the disease. We describe in this work the cloning, expression, purification and characterization of three predicted leptospiral membrane proteins, LIC10258, LIC12880 (Lp30) and LIC12238. We have employed *Escherichia coli* BL21 (SI) strain as a host expression system. Recently, we have identified LIC12238 as a plasminogen (PLG)-binding receptor. We show now that Lp30 and rLIC10258 are also PLG-receptors of *Leptospira*, both exhibiting dose-dependent and saturating binding (*K*_D_, 68.8±25.2 nM and 167.39±60.1 nM, for rLIC10258 and rLIC12880, respectively). In addition, LIC10258, which is a novel OmpA-like protein, binds laminin and plasma fibronectin ECM molecules and hence, it was named Lsa66 (Leptospiral surface adhesin of 66 kDa). Binding of Lsa66 to ECM components was determined to be specific, dose-dependent and saturable, with a *K*_D_ of 55.4±15.9 nM to laminin and of 290.8±11.8 nM to plasma fibronectin. Binding of the recombinant proteins to PLG or ECM components was assessed by using antibodies against each of the recombinant proteins obtained in mice and confirmed by monoclonal anti-polyhistidine antibodies. Lsa66 caused partial inhibition on leptospiral adherence to immobilized ECM and PLG. Moreover, this adhesin and rLIC12238 are recognized by antibodies in serum samples of confirmed leptospirosis cases. Thus, Lsa66 is a novel OmpA-like protein with dual activity that may promote the attachment of *Leptospira* to host tissues and may contribute to the leptospiral invasion. To our knowledge, this is the first leptospiral protein with ECM and PLG binding properties reported to date.

## Introduction

Leptospirosis is a febrile disease caused by pathogenic spirochaetes of the genus *Leptospira*. The disease is been considered an important re-emerging infectious disease [1]. The transmission of leptospirosis has been associated with exposure of individuals in close proximity to wild or farm animals. Lately, the disease became prevalent in cities with sanitation problems and large population of urban rodent reservoirs, which contaminate the environment through their urine [1], [2]. Due to the broad spectrum of symptoms, such as, fever, chills, headache, and myalgias, and similarity with other tropical illness, leptospirosis often remains largely under diagnosed. Progression to multi-organ system complications occurs in 5 – 15% of cases, with mortality rates of 5 – 40% [1], [2]. The disease also has a great economic impact in the agricultural industry since it affects livestock inducing abortions, stillbirths, infertility, reduced milk production and death [1], [2]. Commercially available vaccines, consisting of heat or chemically inactivated leptospires, named bacterins, provide serovar-specific protection against infection. The lack of serovar cross-protection in addition to the need for annual revaccination imposes a major limitation of whole-cell *Leptospira* vaccines [3]. The search for novel protein antigens that could elicit a broad protective and long-term immunity is currently under investigation.

Surface-associated proteins, as a result of their location, are likely to be important in host-pathogen interactions, hence their potential to promote several activities, including adhesion. The interaction of pathogens with the extracellular matrix (ECM) has been well documented [4]. In the case of leptospires, some adhesion ECM-binding molecules have been characterized thus far. These include the Lsa24/Len protein family [5], [6], LigA/LigB [7], Lsa21 [8], LipL32 [9], TlyC [10], Lp95 [11], Lsa63 [12] and OmpL37 [13]. We were the first group to describe that *Leptospira* species are able to bind PLG and generate plasmin, in the presence of activator, on the outer surface *in vitro* [14]. More recently, we have shown that eight out of fourteen recombinant proteins available in our laboratory are PLG-binding proteins [15]. We believe that understanding the molecular mechanism of pathogenesis of *Leptospira* should help in the identification of novel vaccine candidates.

In the present study, we describe the cloning, expression, purification and characterization of three predicted membrane proteins, LIC10258, LIC12880 and LIC12238, identified in the genome sequences of *L. interrogans* serovar Copenhageni [16]. One of them, LIC12238, was previously described as a plasminogen- binding protein [15]. We now show that rLIC12880, previously named Lp30 [17] and rLIC10258 are also plasminogen-receptors of *Leptospira*. In addition, rLIC10258, which is a novel OmpA-like protein, is also capable to bind laminin and plasma fibronectin ECM molecules and hence it was named Lsa66 (Leptospiral surface adhesin of 66 kDa). The recombinant proteins are recognized by antibodies in serum samples of individuals in convalescent phase of the disease. Furthermore, the coding sequences for LIC10258, LIC12880 and LIC12238 may be expressed on the surface of bacteria because they are detected by immunofluorescence assay with intact living leptospires. Thus, it is possible that Lsa66 and LIC12238 proteins are expressed during infection and may play a role in pathogenesis.

## Methods

### *Leptospira* strains and sera

The non-virulent *Leptospira* strains used were: *L. interrogans* serovar Canicola strain Hound Utrech IV, *L. interrogans* serovar Copenhageni strain M 20, *L. interrogans* serovar Icterohaemorrhagiae strain RGA, *L. interrogans* serovar Pomona strain Pomona, *L. interrogans* serovar Hardjo strain Hardjoprajitno, *L. borgpetersenii* serovar Castellonis strain Castellon 3, *L. kirshneri* serovar Grippotyphosa strain Moskva V, *L. santarosai* serovar Shermani strain 1342 K, *L. noguchii* serovar Panama strain CZ 214 and *L. biflexa* serovar Patoc strain Patoc, were cultured at 28°C under aerobic conditions in liquid EMJH medium (Difco®) with 10% rabbit serum, enriched with L-asparagine (wt/vol: 0.015%), sodium pyruvate (wt/vol: 0.001%), calcium chloride (wt/vol: 0,001%), magnesium chloride (wt/vol: 0.001%), peptone (wt/vol:0.03%) and meat extract (wt/vol: 0.02%) [18]. *Leptospira* cultures are maintained in Faculdade de Medicina Veterinária e Zootecnia, USP, São Paulo, SP, Brazil. Confirmed- leptospirosis serum samples were from Instituto Adolfo Lutz collection, São Paulo, Brazil.

### Characterization of the protein *in silico*

Predicted coding sequence (CDS) LIC10258, LIC12880 and LIC12238 were identified using non redundant protein and *L. interrogans* serovar Copenhageni databases the *L. interrogans* serovar Copenhageni [16]; cellular localization prediction was performed by PSORT, http://psort.nibb.ac.jp [19] and CELLO, http://cello.life.nctu.edu.tw/ [20] programs. The SMART, http://smart.embl-heidelbergde/ [21], PFAM, http://www.sanger.ac.uk/Software/Pfam/ [22], and LipoP, http://www.cbs.dtu.dk/services/LipoP/ [23] web servers were used to search for predicted functional and structural domains. Sequence analysis was performed by BLAST [24] using Conserved Domain Database [25].

### DNA isolation and PCR analysis

*Leptospira* cultures were harvested by centrifugation at 11,500 g for 30 min and gently washed in sterile PBS twice. Genomic DNA was isolated from the pellets by guanidine-detergent lysing method using DNAzol® Reagent (Invitrogen), according to the manufacturer's instructions. Primers were designed according to *L. interrogans* serovar Copenhageni genome sequences (GenBank accession AE016823) and are listed in Table 1. PCR was performed in a reaction volume of 25 µl containing 100 ng of genomic DNA, 1× PCR buffer (20 mM Tris-HCl, pH 8.4, 50 mM KCl), 2 mM MgCl_2_, 20 pmol of each specific primer, 200 µM of each dNTP, and 2.5 U Taq DNA Polymerase (Invitrogen). Cycling conditions were: 94°C - 4 min, followed by 40 cycles at 94°C - 50 sec, 64°C (LIC10258) or 56°C (LIC12880) or 58°C (LIC12238) - 50 sec, 72°C - 90 sec, and a final extension cycle of 7 min at 72°C. PCR amplified products were loaded on a 1% agarose gel for electrophoresis and visualization with ethidium bromide.

**Table 1:** Gene locus, protein name, gene bank reference sequence, features, gene conservation, sequence of the primers employed for DNA amplification, and molecular mass of expressed recombinant proteins.

Gene locus^1^	Recombinant protein given name	NCBI reference sequence number^2^	Description/Function	Conservation (identity)^3^	Sequence of primers for PCR amplification	Recombinant protein molecular mass
LIC10258	rLIC10258	YP_000249	Hypothetical protein with ompA domain	Lai (99%); LBH (79%)	F:5′ GGATCC GAAGCCTTCTCACCCAATTG 3′ (BamH I)	65.74 kDa
					R:5′ CCATGGTTAAAGTGAAAGATAAAAATCGATTC 3′ (Nco I)	
LIC12880	Lp30	YP_002796	Putative lipoprotein	Lai (99%); LBH (76%)	F: 5′ CTCGAG GAAGTTGTCCGAGTCTAT 3′ (Xho I)	30.68 kDa
					R: 5′ CCATGG TTATTGATTGTTTAATTCAG 3′ (Nco I)	
LIC12238	rLIC12238	YP_002173	Hypothetical protein	Lai (99%); LBH (77%);	F: 5′ CTCGAG TGTTTTAAACCTACCGGAG 3′ (Xho I)	17.635 kDa
				LBP (39%)	R:5′AAGCTTCTACTTCATCGCTTTTTCTATATC 3′ (Hind III)	

1 http://aeg.lbi.ic.unicamp.br/world/lic/;

2 http://www.ncbi.nlm.nih.gov/protein/;

3 http://www.ncbi.nlm.nih.gov/blast/Blast.cgi/. This work, Lai: *L. interrogans* serovar Lai; LBH: *L. borgpetersenii* serovar Hardjo-bovis; LBP: *L. biflexa* serovar Patoc.

### RNA extraction and RT-PCR analysis

For reverse transcription (RT)-PCR, total RNA was isolated by the acid guanidinium thiocyanate phenol-chloroform method using TRIzol® Reagent (Invitrogen) according to the manufacturer's recommendations. One microgram of RNA from each sample was treated with 1 U of DNAse I Amplification Grade (Invitrogen) for 15 min at room temperature. DNAse I was inactivated by the addition of 1 µl of 25 mM EDTA solution followed by an incubation at 65°C for 10 min. DNAse-treated RNAs were reversely transcribed using the SuperScript™ III First-Strand Synthesis System for RT-PCR (Invitrogen). One tenth of RT products were amplified in a 25 µl reaction mix using oligonucleotides LIC10258-F/LIC10258-R or LIC12880-F/LIC12880-R or LIC12238-F/LIC12238-R as described above. Samples quantity and integrity were verified by amplification of a 1,042 bp 16 S ribosomal cDNA fragment using oligomers:

16F 5′CAAGTCAAGCGGAGTAGCAATACTCAGC 3′ and 16S-R 5′ GATGGCAACATAAGGTGAGGGTTGC 3′.

The effect of temperature on LIC10258 transcript levels within the *L. interrogans* serovar Icterohaemorrhagiae strain was assessed by culturing leptospires at 37°C and 39°C. Additional cultures grown at 30°C were shifted overnight to 37°C and to 39°C, to simulate conditions encountered by bacteria upon entry into the host and in a febrile stage. Induction of LIC10258 expression by osmolarity was examined by centrifugation of cultures grown at 30°C in EMJH supplemented with 1% rabbit serum followed by resuspension in fresh EMJH medium or in EMJH containing 120 mM NaCl. Cultures were incubated for 24 h before being harvested for RNA isolation. The same procedures were performed with LIC12880 in the *L. borgpetersenii* serovar Castellonis, *L. santarosai* serovar Shermani and *L. noguchii* serovar Panama strains.

### DNA recombinant techniques

Predicted CDS LIC10258, LIC12880 and LIC12238, without signal peptides, were amplified by the PCR from *L. interrogans* serovar Copenhageni strain Fiocruz L1–130 genomic DNA using the primer pairs listed in Table 1. The PCR products obtained for each corresponding gene were cloned into pGEM-T easy vector (Promega) and subcloned into the pAE expression vector [26] at the restriction sites depicted in Table 1. The pAE vector allows the expression of recombinant proteins with a minimal 6 x His-tag at the N-terminus. All cloned sequences were confirmed by DNA sequencing with an ABI 3100 automatic sequencer (PE Applied Biosystems, Foster city, CA).

### Expression and purification of recombinant protein

Expression and purification of the recombinant proteins Lp30 and rLIC12238 have been previously described by Neves et al (2007) [17] and Vieira et al (2010) [15], respectively. Protein expression of the Lsa66 was achieved in *E. coli* BL21 (SI) strain by the action of T7 DNA polymerase under control of the osmotically induced promoter proU. *E. coli* BL21 (SI) containing recombinant plasmids were grown at 30°C in Luria-Bertani broth without NaCl and with 100 µg/ml ampicillin with continuous shaking until an optical density at 600 nm of 0.6 to 0.8 was reached. Recombinant protein synthesis was induced by the addition of 30 mM NaCl. After three hours, the cells were harvested by centrifugation, the bacterial pellets resuspended in lysis buffer (10 mM Tris-HCl, pH 8.0, 150 mM NaCl, 100 µg/ml of lysozyme, 1% Triton X-100, 2 mM phenylmethylsulfonyl fluoride [PMSF]). The bacterial cell pellets were lysed on ice with the aid of a sonicator (Ultrasonic Processor; GE Healthcare). The insoluble fraction was washed with 20 ml of buffer (20 mM Tris-HCl, pH 8.0, 500 mM NaCl, 1 M urea and 0,1% Triton X-100) and resuspended in a buffer containing 20 mM Tris-HCl, pH 8.0, 500 mM NaCl, 10% (vol/vol) glycerol and 8 M urea. The protein was then purified through metal chelating chromatography in a Sepharose fast flow column (GE Healthcare) and fractions were analyzed in 12% SDS-PAGE. The protein was extensively dialyzed against phosphate-buffered saline (PBS), pH 7.4, 0.1% (wt/vol) glycine solution (at the proportion of 10 ml of protein per l000 ml of buffer) containing decreasing gradient of glycerol (10 – 0%), pooled and stored at 4°C.

### Circular dichroism spectroscopy

Purified recombinant proteins were dialyzed against sodium phosphate buffer (pH 7.4). Circular dichroism (CD) spectroscopy measurements were performed at 20°C using a Jasco J-810 spectropolarimeter (Japan Spectroscopic, Tokyo) equipped with a Peltier unit for temperature control. Far-UV CD spectra were measured using a 1 mm-path-length cell at 0.5 nm intervals. The spectra were presented as an average of five scans recorded from 180 to 260 nm. The molar ellipticity (Φ) is expressed in deg.cm. dmol^−1^. Spectra data was submitted to DICROPROT web server, http://dicroprot-pbil.ibcp.fr/, using the method that calculated the secondary structure content from the ellipticity experimental data [27].

### Antiserum

Five female BALB/c mice (4 – 6 weeks old) were immunized subcutaneously with 10 µg of the recombinant proteins. The recombinant protein was adsorbed in 10% (vol/vol) of Alhydrogel (2% Al(OH)_3_, Brenntag *Biosector*, Denmark), used as adjuvant. Two subsequent booster injections were given at two-week intervals with the same preparation of 10 µg of the proteins. Negative-control mice were injected with PBS. One week after each immunization, the mice were bled from the retro-orbital plexus and the pooled sera were analyzed by enzyme-linked immunosorbent assay (ELISA) for determination of antibody titers. All animal studies were approved by the Ethics Committee of the Instituto Butantan, São Paulo, SP, Brazil under protocol n^º^ 471/08. The Committee in Animal Research in Instituto Butantan adopts the guidelines of the Brazilian College of Animal Experimentation.

### Immunoblotting assay

The purified recombinant proteins were loaded into 15% SDS-PAGE and transferred to nitrocellulose membranes (Hybond ECL; GE Healthcare) in semi-dry equipment. Membranes were blocked with 5% non-fat dried milk and 2.5% BSA in PBS containing 0.05% Tween 20 (PBS-T) and then incubated with anti-Lsa66 (1∶800), anti-Lp30 (1∶800), anti-rLIC12238 (1∶2,000) mouse serum or anti-his antibody (1∶3,000) (GE Healthcare) for 2 h at room temperature. After washing, the membrane was incubated with horseradish peroxidase (HRP)-conjugated anti-mouse IgG (1∶5,000; Sigma) in PBS-T for 1 h. The proteińs reactivity was revealed by ECL reagent kit chemiluminescence substrate (GE Healthcare) with subsequent exposition to X-Ray film.

### Microscopic agglutination test (MAT)

The microscopic agglutination test was performed according to Faine et al. (1999) [1]. In brief, an array of serovars of *Leptospira* spp. as antigens were employed: Australis, Autumnalis, Bataviae, Canicola, Castellonis, Celledoni, Copenhageni, Cynopteri, Djasiman, Grippotyphosa, Hardjo, Hebdomadis, Icterohaemorrhagiae, Javanica, Panama, Patoc, Pomona, Pyrogenes, Sejroe, Shermani, Tarassovi and Wolffi. All the strains were maintained in EMJH liquid medium (Difco, USA) at 29°C. A laboratory-confirmed case of leptospirosis was defined by the demonstration of a four-fold microagglutination titer rise between paired serum samples. The probable predominant serovar was considered to be the one with the highest dilution that could cause 50% of agglutination. MAT was considered negative when the titer was below 100.

### ELISA for detection of human antibodies

Human IgG and IgM antibodies against Lsa66, Lp30 and rLIC12238 were evaluated by ELISA. In brief, samples (negative and positive MAT sera from 10 confirmed leptospirosis patients) were diluted 1∶100 and evaluated for total IgG and IgM using peroxidase-conjugated anti-human IgG and IgM antibodies, 1∶5,000 (Sigma, USA). Cutoff values were set at three standard deviations above the mean OD_492_ of sera from 6 individuals, unexposed to leptospirosis, from the city of São Paulo, Brazil.

### Plasminogen binding assay

The binding of the recombinant proteins to PLG was evaluated by a modified ELISA as follows: 96-well plates (Costar High Binding, Corning) were coated overnight in PBS at 4°C with 100 µl of 10 µg/ml of the human plasminogen; fetuin and bovine serum albumin (BSA) were employed, as negative control. Plates were washed once with PBS supplemented with 0.05% (vol/vol) Tween 20 (PBS-T) and blocked for 2 h at 37°C with PBS with 10% (wt/vol) non-fat dry milk. The blocking solution was discarded and 100 µl of 10 µg/ml recombinant proteins in PBS was incubated for 2 h at 37°C. Wells were washed four times with PBS-T and incubated for 1 h at 37°C with polyclonal mouse anti-recombinant proteins (1∶1,000 in PBS). In another assay, anti- His tag monoclonal antibodies (Sigma) were employed to detect protein binding at 1∶1000 dilution. Plates were washed again and incubated with horseradish peroxidase-conjugated anti-mouse immunoglobulin G (IgG), diluted 1∶5,000 in PBS. After three washings, 100 µl/well of 1 mg/ml *o*-phenylenediamine (OPD) plus 1 µl/ml H_2_O_2_ in citrate phosphate buffer (pH 5.0) were added. The reactions were carried out for 5 min and stopped by the addition of 50 µl/well of H_2_SO_4_ (2 N). Readings were taken at 492 nm.

### Plasmin enzymatic activity assay

96-well ELISA plates were coated overnight with 10 µg/ml recombinant proteins (or BSA for negative control) in PBS at 4°C. Plates were washed once with PBS-T and blocked for 2 h at 37°C with PBS with 10% (wt/vol) non-fat dry milk. The blocking solution was discarded and 100 µl/well of 10 µg/ml human plasminogen was added, followed by incubation for 2 h at 37°C. Wells were washed three times with PBS-T, and then 4 ng/well of human uPA (Sigma-Aldrich) was added. Subsequently, 100 µl/well of plasmin-specific substrate _D_-valyl-leucyl-lysine-*p*-nitroanilide dihydrochloride (Sigma- Aldrich) was added at a final concentration of 0.4 mM in PBS. Plates were incubated overnight at 37°C and substrate degradation was measured by taken the readings at 405 nm.

### Binding of recombinant proteins to ECM

Protein attachment to individual macromolecules of the extracellular matrix was analyzed according to a previously published protocol [6] with some modifications. Briefly, ELISA plate wells were coated with 1 µg of laminin, collagen type I, collagen type IV, cellular fibronectin, plasmatic fibronectin, BSA (nonglycosylated attachment-negative control protein) and fetuin (highly glycosylated attachment-negative control protein) in 100 µL of PBS for 2 h at 37°C. The wells were washed three times with PBS containing 0.05% Tween 20 and then blocked with 200 µL of 1% BSA (overnight at 4°C). One microgram of each protein (Lsa66∼152 nM, Lp30∼326 nM and rLIC12238∼567 nM) was added per well in 100 µL of PBS, and proteins were allowed to attach to the different substrates for 2 h at 37°C. After washing six times with PBS-T, bound proteins were detected by adding an appropriate dilution of mouse antiserum in 100 µL of PBS. Dilutions of mouse antiserum against each recombinant protein were equalized as to give an OD_492 nm_ value of 1.0, as follows: Lsa66 (LIC10258) 1∶800; Lp30 (LIC12880) 1∶10,000 and rLIC12238 (LIC12238) 1∶10,000. In addition, anti-polyhistidine monoclonal antibodies were employed as protein-binding probes at 1∶1000 dilution. Incubation proceeded for 1 h at 37°C, and after three washings with PBS-T, 100 µL of a 1∶5,000 dilution of horseradish peroxidase-conjugated goat anti-mouse IgG in PBS was added per well for 1 h at 37°C. The wells were washed three times, and *o*-phenylenediamine (1 mg/mL) in citrate phosphate buffer (pH 5.0) plus 1 µL/mL H_2_O_2_ was added (100 µL per well). The reaction was allowed to proceed for 10 min and was then interrupted by the addition of 50 µL of 8 M H_2_SO_4_. The absorbance at 492 nm was determined in a microplate reader (Multiskan EX; Labsystems Uniscience). For determination of dose-dependent attachment of Lsa66 to laminin and plasma fibronectin, protein concentrations varying from 0 to 1,500 nM in PBS were used. For statistical analyses, the binding of Lsa66 to ECM macromolecules was compared to its binding to BSA and fetuin by 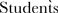 two-tailed *t* test.

### Dissociation constant (*K*_D_) for the recombinant proteins binding to ECM and PLG

The ELISA data were used to calculate the dissociation constant (*K*_D_) according to the method previously described [28] based on the equation: A =  Amax [protein]/(*K*_D_ + [protein]), where A is the absorbance at a given protein concentration, Amax is the maximum absorbance for the ELISA plate reader (equilibrium), [protein] is the protein concentration and *K*_D_ is the dissociation equilibrium constant for a given absorbance at a given protein concentration (ELISA data point).

### Inhibition of live leptospires binding to laminin, plasma fibronectin and plasminogen by Lsa66 and Lp30

ELISA plates were coated with laminin or plasma fibronectin or plasminogen (1 µg/well). The plates were washed and blocked with 10% non-fat dry milk in PBS-T for 2 h at 37°C. The blocking solution was discarded, and the wells were incubated for 90 min at 37°C with increasing concentrations of recombinant proteins (0 to 5.0 µg). After three washings, 100 µL/well of 4×10^7^ live *L. interrogans* serovar Copenhageni strain M20 were added for 90 min at 37°C. The unbound leptospires were washed and the quantification of bound leptospires was performed indirectly by anti-LipL32 antibodies produced in mice (1∶4,000), based on the fact that LipL32 is a major outer membrane leptospiral protein [29]; the procedure was followed by horseradish peroxidase-conjugated anti-mouse IgG antibodies, essentially as described in Barbosa et al. (2006) [6]. The detection was performed by OPD as above described.

### Liquid-phase immunofluorescence assay (L-IFA)

The localization of LIC10258, LIC12880 and LIC12238 encoded proteins by L-IFA was performed as described Oliveira et al (2010) [30]. In brief, suspensions of 2.5 ml live leptospires (∼10^9^cells/ml) were harvested at 10,000 rpm for 15 min, washed twice with PBS (with 50 mM NaCl), resuspended in 200 µl of PBS with 6 µg/ml propidium iodide to stain the nuclei, and incubated for 45 min at 37°C. After incubation, the leptospires were washed gently with PBS and incubated for 30 min at 4°C with polyclonal mouse anti-serum against Lsa63, rLipL32 or rGroEL at a 1∶50 dilution. The leptospires were washed and incubated with goat anti-mouse IgG antibodies conjugated to fluorescein isothiocyante (FITC, Sigma) at a dilution 1∶50 for 30 min at 4°C. After incubation with secondary antibody, the leptospires were washed and resuspended in PBS-antifading solution (ProLong Gold, Molecular Probes). The immunofluorescence-labeled leptospires were examined by use of a confocal LSM 510 META immunofluorescence microscope (Zeiss, Germany).

### Statistical analysis

All results are expressed as means ± SEM. Student's paired *t* test was used to determine the significance of differences between means, and P lower than 0.05 was considered as statistically significant.

### ECM components and plasminogen

All macromolecules, including the control proteins fetuin and BSA, were purchased from Sigma Chemical Co. (St. Louis, Mo.). Laminin-1 and collagen Type IV were derived from the basement membrane of Engelbreth-Holm-Swarm mouse sarcoma, cellular fibronectin was derived from human foreskin fibroblasts, plasma fibronectin was isolated from human plasma and collagen Type I was isolated from rat tail. Plasminogen native, purified from human plasma, was purchased from Merck.

### Nucleotide sequence accession numbers

Gene bank accession number for protein sequences LIC10258, LIC12880 and LIC12238 is AAS68886, AAS71433 and ASS70810, respectively (see Table 1). The protein can also be accessed by the genome nomenclature for the gene locus, LIC number (*Leptospira interrogans* Copenhageni).

## Results

### Bioinformatic analysis

**Figure 1:**
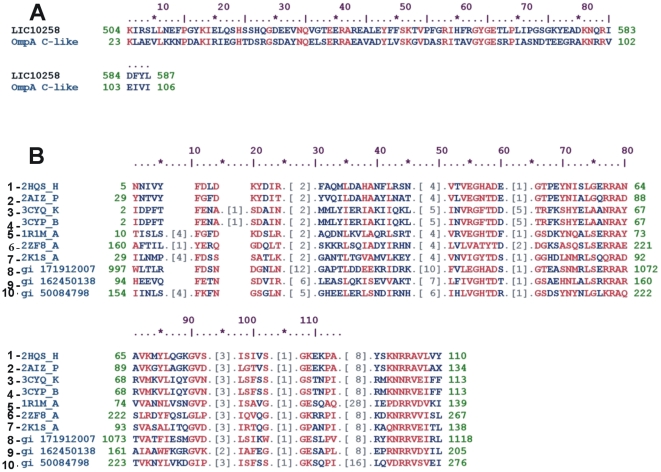
Sequence analysis. (A) Sequence alignment of the OmpA C-like domain with the C-terminal region of CDS LIC10258. (B) Sequence alignments comparison of C-terminal region of CDS LIC10258 with proteins having OmpA-like domain from diverse pathogens: 1. *Escherichia coli* (chain H); 2. PAL of *Haemophilus influenza*; 3. *Helicobacter pylori* (chain K); 4. *Helicobacter pylori* (chain B); 5. *Neisseria meningitides*; 6. *Vibrio alginolyticus*; 7. *Escherichia coli* (chain A); 8. *Verrucomicrobium spinosum* DSM 4136; 9. *Sorangium cellulosum*; 10. *Acinetobacter* sp. ADP1 (putative antigen).

The genes encoding LIC10258, LIC12880 and LIC12238 were identified by analysis of the genome sequences of the chromosome I of *L. interrogans* serovar Copenhageni and each one is present as a single copy [16]. The CDSs LIC10258, LIC12880 and LIC12238 have signal peptide ranging from amino acid 1 to 28, according to SMART web server, and are predicted to be outer membrane proteins based PSORT [19] and CELLO [20] web servers. The LIC12280 and LIC12238 are putative lipoproteins, with a cleavage site for signal peptidase II, according to LipoP program [23]. Blast analysis showed that the CDS LIC10258 has a putative OmpA- like domain at the C-terminal, from amino acid 504 to 583. Sequence alignment of the OmpA C-like domain with the C-terminal region of LIC10258 is depicted in Fig. 1A. Sequence alignments of OmpA-like conserved domain from diverse pathogens [25] are depicted in fig. 1B. The LIC10258 is present in the genome sequences of *L. interrogans* serovar Lai (99% identity) [31], of *L. borgpetersenii* serovar Hardjo-bovis (79% identity) [32], but is absent in *L. biflexa* serovar Patoc [33]. Similar putative coding sequence of LIC12880 was found in *L. interrogans* serovar Lai (99% identity), in *L. borgpetersenii* serovar Hardjo-bovis (76% identity), but is absent in *L. biflexa* serovar Patoc. The CDS LIC12238 was identified in *L. interrogans* serovar Lai (99% identity), in *L. borgpetersenii* serovar Hardjo-bovis (77% identity) and in *L. biflexa* serovar Patoc (39% identity). Table 1 summarizes the main features of the selected proteins and gene conservation within the sequenced genomes [31], [32], [33].

### Distribution and expression of LIC10258, LIC12880 and LIC12238 genes among *Leptospira* strains

**Figure 2:**
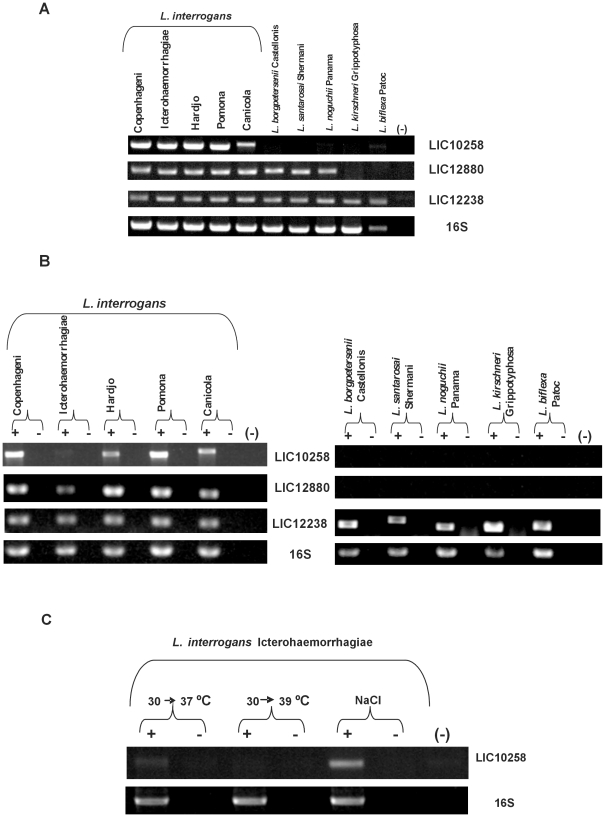
Distribution and expression of LIC10258, LIC12880 and LIC12238 genes in saprophytic and pathogenic leptospires. (A) Genomic DNA from *L. biflexa* Patoc and from nine serovars belonging to the pathogenic species of *Leptospira* were subjected to PCR analysis with specific primers designed according to *L. interrogans* serovar Copenhageni genome sequences. Amplification of 16S DNA shows template integrity. No DNA was added to the negative control reaction (-). (B) RT-PCR analysis of LIC10258, LIC12880 and LIC12238 transcripts in high-passage *Leptospira* strains. Reactions were performed with specific primers designed according *L. interrogans* serovar Copenhageni. Samples quantity and integrity were verified by amplification of 16S ribosomal cDNA fragment. +: reverse transcriptase present. -: reverse transcriptase omitted. No cDNA was added to the negative control reaction (-). (C) Transcript analysis of LIC10258 in *L. interrogans* serovar Icterohaemorrhagiae after submission of bacterial culture to temperature upshift from 30 to 37°C, 30 to 39°C and to physiological osmmolarity.

The LIC10258 gene is present in the main serovars of *L. interrogans*, Copenhageni, Icterohaemorrhagiae, Hardjo, Pomona and Canicola but no DNA amplification was detected in *L. borgpertesenii* serovar Castellonis, *L. santarosai* serovar Shermani, *L. noguchii* serovar Panama, *L. kirschneri* serovar Grippotyphosa and in *L. biflexa* serovar Patoc (Fig.2A). Gene LIC12880 was found in all serovars tested of *L. interrogans* and in *L. borgpertesenii* serovar Castellonis, *L. santarosai* serovar Shermani, *L. noguchii* serovar Panama, but absent in *L. kirschneri* serovar Grippotyphosa and in *L. biflexa* serovar Patoc (Fig. 2A). Also shown in this figure is the DNA conservation of LIC12238 that was identified in all tested strains. 16S DNA amplification was performed to attest template integrity (Fig. 2A). The expression of LIC10258, LIC12880 and LIC12238 by *in vitro* cultured leptospires was evaluated by PCR amplification of reversely transcribed total RNA. The results obtained revealed the presence of LIC10258 transcripts in four *L. interrogans* strains mentioned above but no amplification was detected with serovar Icterohaemorrhagiae (Fig. 2B). In the case of LIC12880, transcripts were observed only in serovars of *L. interrogans*, although the gene was found in other pathogenic strains (Fig. 2A e 2B). Transcripts of LIC12238 were identified in all analyzed strains (Fig. 2B). DNA contamination was discarded as no amplification was observed in the absence of reverse transcriptase. The integrity of total RNA used in RT-PCR experiment was assured by the presence of a 1,042-bp 16S ribosomal cDNA fragment in all samples (Figure 2B). We set out to examine whether environmental factors, such as osmolarity and temperature could influence LIC10258 and LIC12880 regulation at the transcriptional level. Induction of LIC10258 and LIC12880 expression by osmolarity was assessed by growing cultures at 30°C in EMJH supplemented with 1% rabbit serum and resuspending them in fresh EMJH medium or in EMJH containing 120 mM NaCl. The addition of 120 mM NaCl to the medium mimics physiological conditions (∼300 mosmol per liter) encountered by leptospires upon entry into the host [34]. We also evaluated gene expression of both genes from cultures subjected to temperature upshifts from 30°C to 37°C and from 30°C to 39°C during an overnight period to simulate conditions experienced by leptospires in the early stages of infection and during febrile stage. The results obtained show that cultures of *L. interrogans* serovar Icterohaemorrhagiae submitted to temperature upshift from 37°C to 39°C had no effect on the level of LIC10258 transcript (Fig. 2C). However, LIC10258 transcription (Fig. 2C) could be detected when the same culture strain was shifted to physiological osmolarity, at 30°C. In the case of LIC12880, no transcripts were observed in *L. borgpertesenii* serovar Castellonis, *L. santarosai* serovar Shermani and *L. noguchii* serovar Panama culture strains submitted to the same temperature and osmolarity conditions above described (data not shown).

### Expression and purification of recombinant proteins

**Figure 3:**
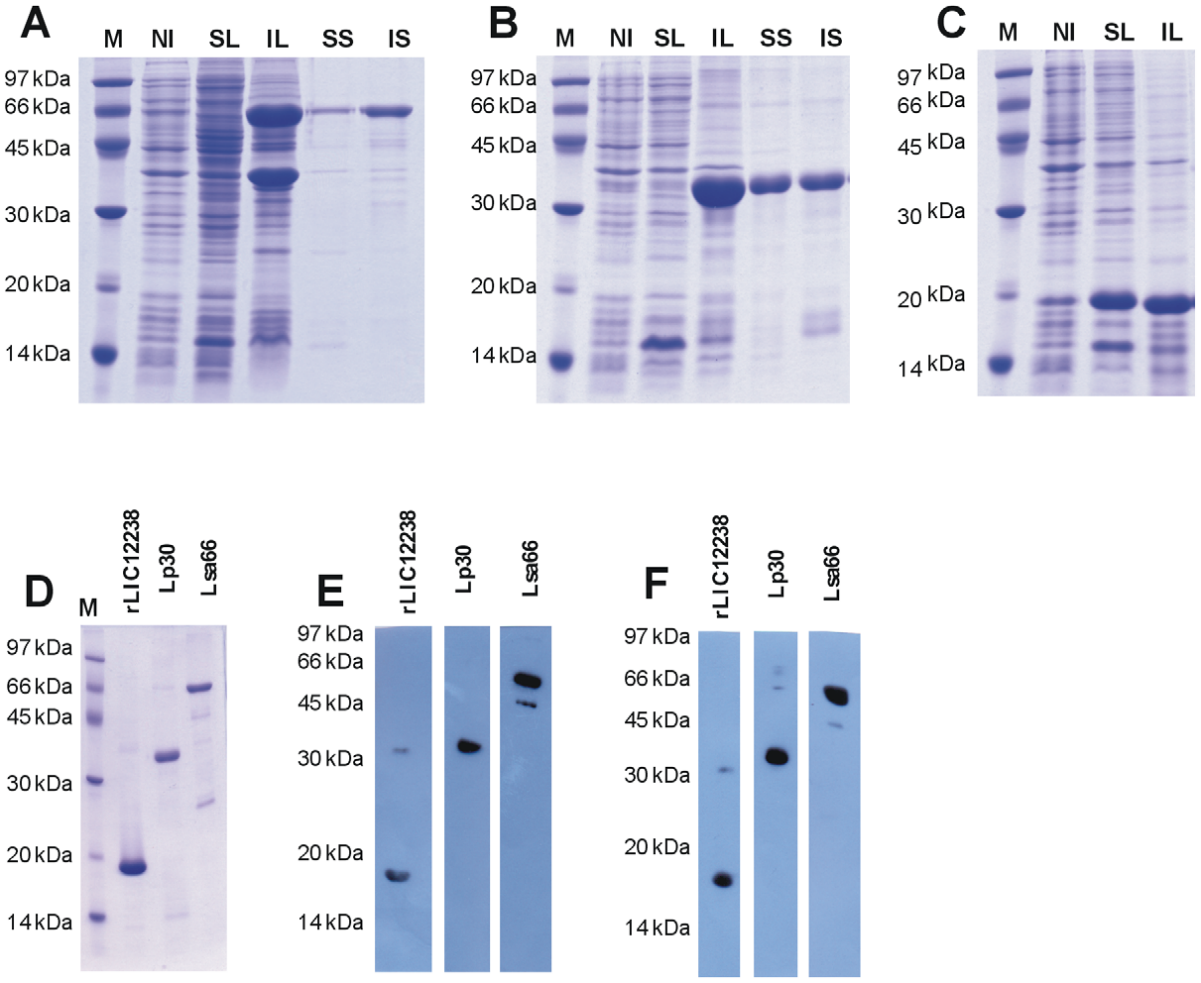
Protein analysis by SDS-PAGE and Western blotting. (A) Lsa66, (B) Lp30 and (C) rLIC12238 expression from NaCl-induced *E. coli* Bl21-SI. M: molecular mass protein marker; NI: non-induced total bacterial extract; SL: supernatant after bacterial cell lysis and centrifugation; IL: inclusion body pellet after bacterial lysis and centrifugation; SS: soluble fraction of the induced culture in the presence of 8 M urea; IS: insoluble fraction of the induced culture in the presence of 8 M urea. (D) Comassie blue stained purified recombinant proteins. (E) and (F) are western blotting analysis of the recombinant proteins probed with anti-His tag antibodies and the respective homolog antiserum, respectively.

The amplified coding sequences, excluding the signal peptide tags, were cloned and expressed as full-length proteins in *E. coli*. Gene locus, protein reference number, given name, sequences of primers used for PCR amplifications and molecular mass of recombinants are illustrated in Table 1. The recombinant proteins were purified by nickel affinity chromatography, and an aliquot of each protein was analyzed by SDS-PAGE (Fig. 3A–C). All purified proteins were represented by major bands (Fig. 3D). To further confirm that these proteins are indeed his-tag recombinants we carried out immunoblotting technique and probed them with his-tag antibodies. The data is shown in figure 3E and, as we can see, anti-His tag antibodies recognized the 3 recombinant proteins. However, in the case of rLIC12238 and Lsa66 preparation, other protein bands were also detected, being in the first case probably due to non-specific reaction and in the second, possibly caused by some protein degradation. Similar data were obtained when blotted recombinant proteins were probed with the respective homolog antiserum raised in mice (Fig. 3F).

### Structural integrity of the purified proteins was assessed by circular dichroism (CD) spectroscopy

**Figure 4:**
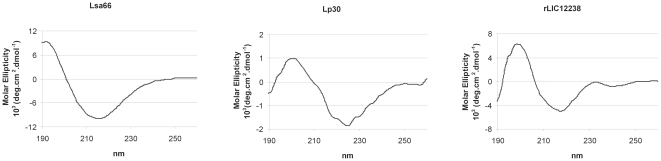
Circular dichroism spectra of the recombinant proteins. CD spectra of recombinant proteins Lsa66, Lp30 and rLIC12238. Far-UV CD spectra are presented as an average of five scans recorded from 190 to 260 nm. Experimental conditions, as described in M&M.

As depicted in Fig. 4, the maximum at 192 nm of α–helix with a broad minimum ellipticity around 215 nm, probably of β-strands, in the CD spectrum show that Lsa66 has both secondary structures; maximum ellipticity of 198 nm of β-strands was detected with rLIC12238, while maximum ellipticity around 200 nm was observed with the protein Lp30. According to DICROPROT web server, the α–helix percentage of the proteins was higher in Lsa66 followed by rLIC12238, being the lower value for Lp30. These results are consistent with a mixture of α–helices and β-strands and are in agreement with the *in silico* analysis that showed both secondary structures (http://bioinf.cs.ucl.ac.uk/psipred/).

### Detection of the LIC10258, LIC12880 and LIC12238 coding sequences by immunofluorescence confocal microscopy

**Figure 5:**
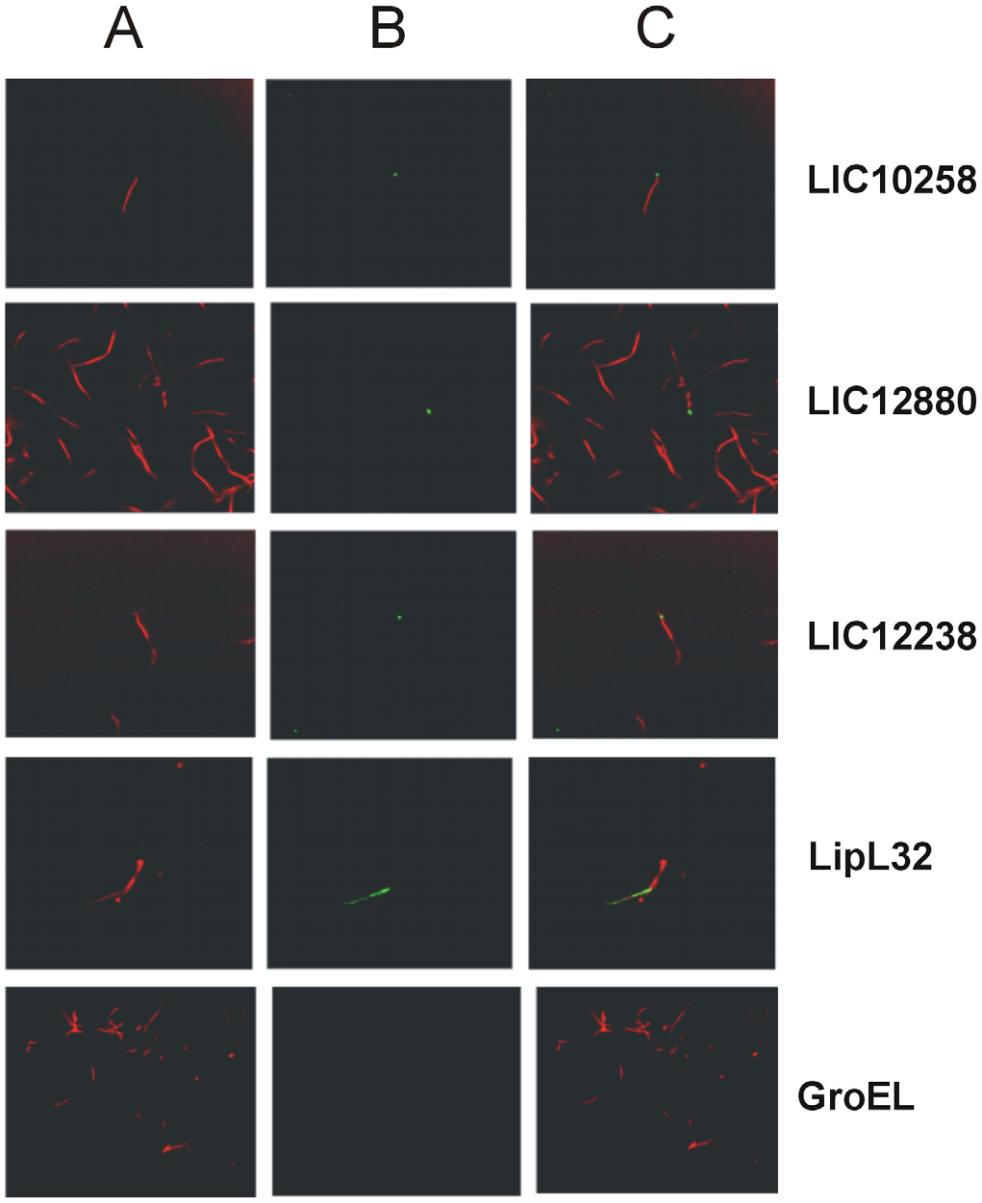
Localization of proteins in *L. interrogans* by L-IFA. Confocal microscopy was performed with live *L. interrogans* using antisera specific for LIC10258, LIC12880, LIC12238, LipL32 (surface-exposed lipoprotein) and GroEL (protoplasmic cylinder marker). FITC-conjugated secondary antibodies were used to detect the surface-bound antibodies (B). Leptospires were identified by propidium iodide (A) staining of the DNA. Co-localization is shown in the merged images (C).

To evaluate whether the selected CDSs are located at the bacterial membrane, we set out to analyze the protein position by using living organisms and the liquid-phase immunofluorescence method. Leptospires were visualized by propidium iodide staining (Fig. 5, column A) followed by protein detection with polyclonal mouse antiserum raised against the protein in the presence of anti-mouse IgG antibodies conjugated to FITC. Green fluorescence could be observed in figure 5 column B, for Lsa66, Lp30, rLIC12238 and LipL32, an outer membrane protein used as a positive control [29], but not with GroEL, a protoplasmic-cylinder marker, used as a negative control [35]. The localization of the protein-green light lying on the leptospires was achieved by merging both fields and the results obtained are shown in figure 5, column C.

### Recombinant leptospiral proteins bind human plasminogen

**Figure 6:**
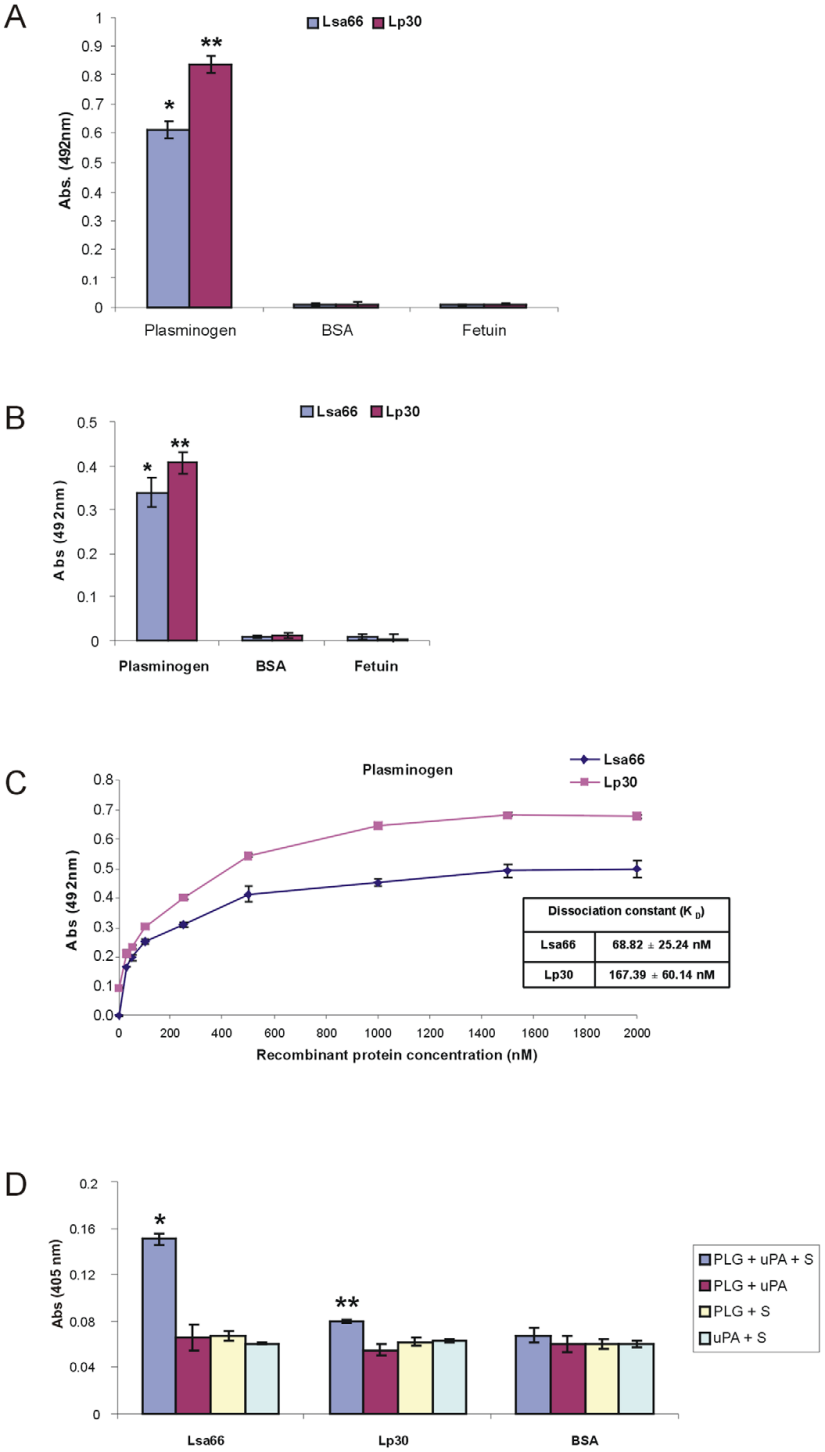
Recombinant proteins binding to human plasminogen. (A) Binding of leptospiral recombinant proteins to human plasminogen. Human purified plasminogen (10 µg/ml) was coated to ELISA plates and allowed to interact with the recombinant proteins Lsa66 and Lp30 (10 µg/ml). BSA and fetuin were used as a negative control for nonspecific binding. The binding was detected by specific antibodies. Bars represent the mean of absorbance at 492 nm ± the standard deviation of three replicates for each protein and are representative of three independent experiments. For statistical analyses, the binding of Lsa66 and Lp30 to human PLG was compared to its binding to BSA as well as fetuin by two-tailed *t* test (*P<0.005 and **P<0.0005). (B) Similar as described in (A) but the binding of the recombinant proteins was detected by specific anti-polyhistidine monoclonal antibodies. (C)Ten µg/ml pf PLG was immobilized into 96-wells ELISA plates and 0 to 2,000 nM of each recombinant protein was added for interaction. The binding was detected using antiserum raised in mice against each protein in appropriate dilutions (1∶500 for Lsa66 and 1∶500 for Lp30) followed by horseradish peroxidase-conjugated anti-mouse IgG. Data represent the mean absorbance values ± the standard deviation of three replicates for each experimental group. The dissociation constant (*K*_D_) was calculated based on ELISA data for the recombinant proteins that have reached the equilibrium concentration. (D) Plasmin generation by PLG bound to recombinant proteins was assayed by modified ELISA as immobilized proteins received the following treatment: PLG + uPA + specific plasmin substrate (PLG + uPA + S), or controls lacking one of the three components (PLG + uPA; PLG + S; uPA + S). BSA was employed as negative control. Bars represent mean absorbance at 405 nm, as a measure of relative substrate degradation ± the standard deviation of four replicates for each experimental group and are representative of two independent experiments. Statistically significant binding in comparison to the negative control (BSA) are show: *P<0.001 and **P<0.02.

We have reported that leptospires bind PLG and that several proteins could act as receptors, including the recombinant protein rLIC12238 of this work [14], [15]. We then decided to investigate whether the selected surface-exposed proteins, Lp30 and Lsa66, were also capable of binding human PLG *in vitro*. Human purified plasminogen was coated to ELISA plates, allowed to interact with the recombinant proteins Lsa66 and Lp30 and the results obtained from three independent experiments using antibodies against the recombinant proteins are shown in Fig. 6A. The binding of the proteins were also evaluated with anti-polyHis monoclonal antibodies (Fig. 6B). The interactions between the recombinant proteins and PLG were assessed on a quantitative basis as illustrated in Fig. 6C. Dose-dependent and saturable binding was observed when increasing concentrations (0 to 2,000 nM) of the recombinant proteins Lsa66 and Lp30 were allowed to individually adhere to a fixed PLG amount (1 µg). Based on the ELISA data, the calculated dissociation equilibrium constants (*K*_D_) for the recombinant proteins with PLG is 68.8±25.2 nM and 167.39±60.1 nM, for Lsa66 and Lp30, respectively (Fig. 6C).

### Plasmin generation from PLG-bound proteins

PLG bound to the surface of *L. interrogans* is converted to enzymatically active plasmin by the addition of activator [14]. To evaluate if PLG bound to recombinant proteins can acquire proteolytic activity, as reported for rLIC12238 [15], 96-well plates were coated with the test proteins, blocked, and then incubated with PLG. Unbound PLG was washed away and the uPA-type PLG activator was added together with a plasmin-specific chromogenic substrate. The reaction was carried out overnight and the plasmin activity was evaluated by measuring the cleavage of the plasmin-specific chromogenic substrate (absorbance at 405 nm). As shown in Fig. 6D, PLG captured by the proteins could be converted into plasmin, as indirectly demonstrated by specific proteolytic activity. The negative control BSA did not bind PLG (see Fig. 6A) and did not show any proteolytic activity. The same situation occurred with the controls lacking PLG, uPA or the chromogenic substrate.

### Adhesion of recombinant proteins to ECM components

**Figure 7:**
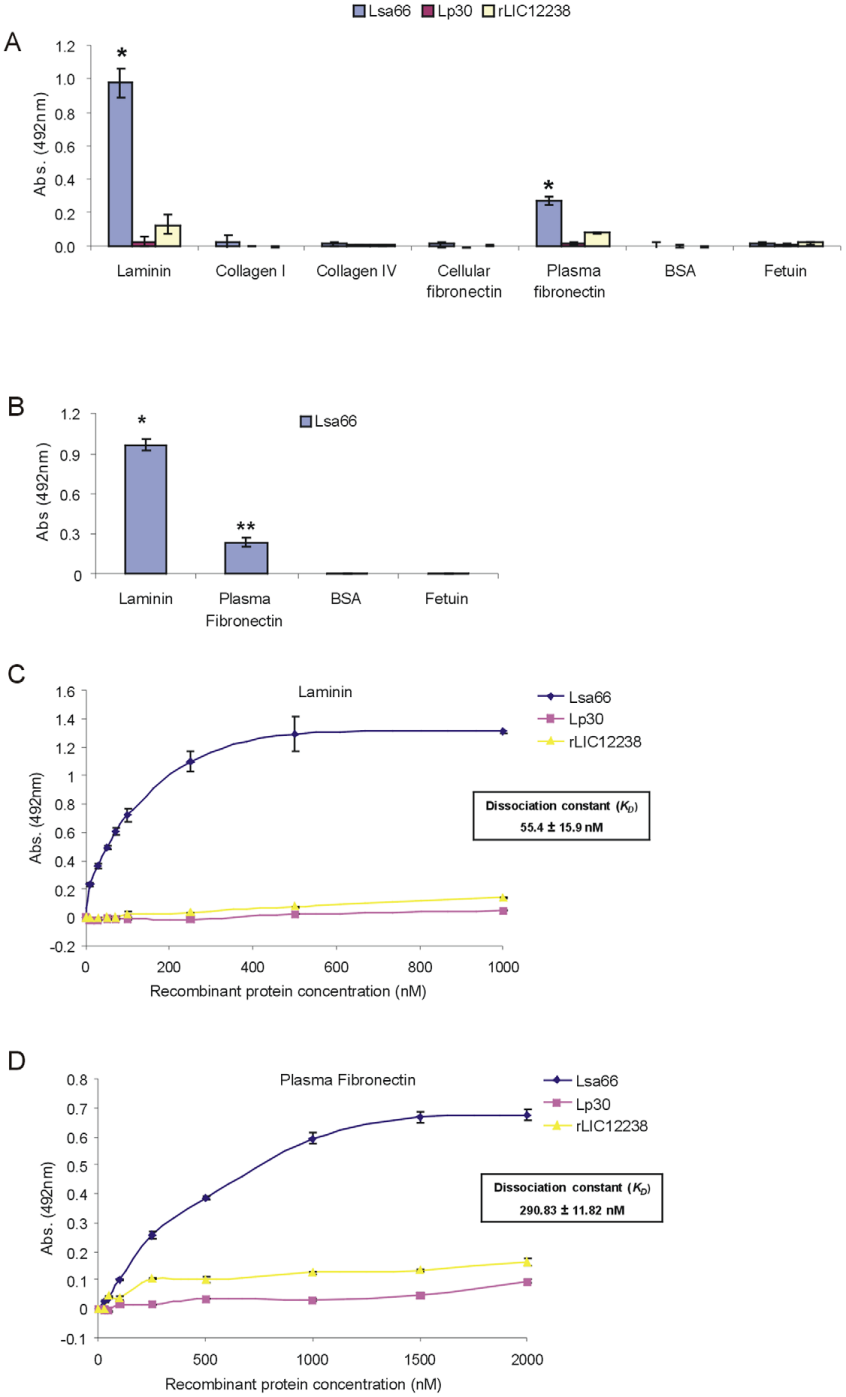
Binding characteristics of Lsa66, Lp30 and rLIC12238 to ECM components. (A) Wells were coated with 1 µg of laminin, collagen type I, collagen type IV, cellular fibronectin, plasma fibronectin and the control proteins BSA and fetuin. 1 µg of the recombinant protein was added per well and the binding was measured by an ELISA-based assay. In (A) the protein binding was detected by polyclonal antibodies against each protein, while in (B) protein binding was evaluated by anti-polyhistidine monoclonal antibodies. Data represent the mean ± the standard deviation from three independent experiments. For statistical analyses, the attachment of recombinant proteins to the ECM components was compared to its binding to BSA as well as fetuin by the two-tailed *t* test (*P<0.005). (C) and (D) are recombinant proteins dose-dependent binding experiments with laminin (C) and plasma fibronectin (D); in both cases bindings were detected by polyclonal antibodies against each protein; each point was performed in triplicate and expressed as the mean absorbance value at 492 nm ± standard error for each point. Lp30 and rLIC12238 were included as a negative control. The dissociation constants (*K*_D_) are depicted and were calculated based on ELISA data for the recombinant proteins that reached equilibrium up to a concentration of 1,000 nM, in the case of laminin and 2,000 nM, for the plasma fibronectin.

The Lsa66, Lp30 and rLIC12238 proteins are suggested to be surface-exposed by immunofluorescence microscopy. We therefore investigated whether these proteins could mediate host colonization by adhering to extracellular matrix proteins. Thus, laminin, collagen Type I, collagen Type IV, cellular fibronectin, plasma fibronectin, and the control protein fetuin were immobilized on microdilution wells and recombinant protein attachment was assessed by an ELISA-based assay using antibodies against the proteins. As shown in Fig. 7A, Lsa66 protein exhibited efficient adhesiveness to laminin and plasma fibronectin. No statistically significant adhesiveness was observed with this protein when wells were coated with collagen Type I and IV, plasma fibronectin or with the highly glycosylated control protein fetuin. No binding was observed with the proteins Lp30 and rLIC12238 with ECM components (Fig. 7A). The Lsa66 interaction with ECM molecules was also observed when anti-polyhistidine monoclonal antibodies were employed (Fig. 7B). The interaction between Lsa66 with laminin and with plasma fibronectin was also assessed on a quantitative basis as depicted in Fig. 6B and 6C, respectively. A dose-dependent and saturable binding was observed when increasing concentrations of the recombinant protein (0 – 1000 nM) were allowed to adhere to a fixed laminin concentration (1 µg) (Fig. 7C) or recombinant protein (0– 2000 nM) to plasma fibronectin (1 µg) (Fig. 7D). Binding saturation level was reached by protein concentration of ∼500 nM, in the case of laminin, and above 1500 nM for plasma fibronectin (Fig. 7C and 7D), respectively. Also shown in these figures are the calculated dissociation equilibrium constants (*K*_D_) for the recombinant protein Lsa66 with laminin, 55.4±15.9 nM and with plasma fibronectin, 290.8±11.8 nM.

### Inhibition of *L. interrogans* attachment to ECM and plasminogen by Lsa66 and Lp30

**Figure 8:**
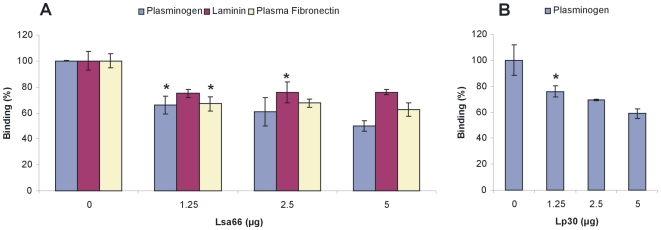
Inhibition of *L. interrogans* attachment to ECM and plasminogen by Lsa66 and Lp30. ECM- or plasminogen-coated microtiter wells were incubated with increasing concentration (0–5.0 µg) of Lsa66 (**A**) or plasminogen-coated microtiter wells and increasing concentration (0–5.0 µg) of Lp30 (**B**) for 1 h30 min prior to the addition of 4×10^7^ leptospires. Wells were probed with anti-LipL32 serum. Data are expressed as A_492 nm_ ± S.E. of three independent experiments, each performed in triplicate. Significance was assessed by comparison with the “no protein” wells by Students two-tailed *t* test (*, *P*<0.05).

The inhibitory effect promoted by Lsa66 on leptospiral adherence to laminin, plasma fibronectin and by Lsa66 and Lp30 to plasminogen was quantified by an ELISA-like assay. ECM- and plasminogen-coated microtiter wells were incubated with increasing concentration of Lsa66 (Fig. 8A) and Lp30 (Fig. 8B) previous to the addition of 4×10^7^ *L. interrogans*. Bound leptospires were probed with anti-LipL32 serum, as LipL32 is a major outer membrane leptospiral protein [29]. Lsa66 caused an inhibition of leptospires attachment to laminin (*P*<0.05), to plasma fibronectin (*P*<0.05) and to plasminogen (*P*<0.05) (Fig. 8A). In the case of Lp30, inhibition of leptospiral adherence to plasminogen was achieved with 1.25 µg (*P*<0.05) (Fig. 8B). The experiment was performed in triplicate and repeated three times with similar results.

### Reactivity of recombinant proteins with sera from confirmed cases of leptospirosis

**Figure 9:**
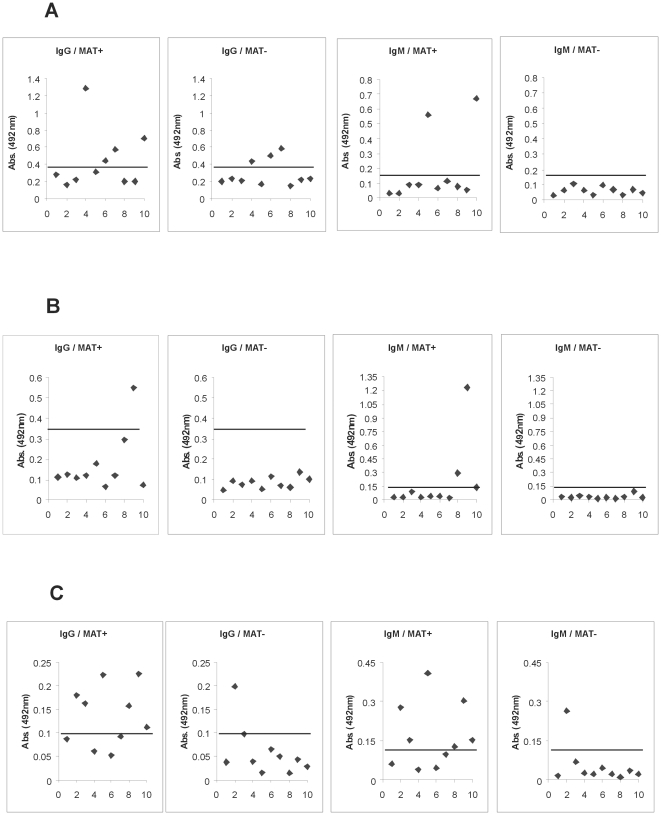
Reactivity of the recombinant antigens Lsa66, Lp30 and rLIC12238 with serum samples of individuals diagnosed with leptospirosis. Positive sera (responders) were determined by ELISA with the recombinant proteins and serum samples from patients in both phases of the disease. The reactivity was evaluated as IgM or IgG antibodies. Serum was considered MAT positive or MAT negative if agglutination was detected when the sera were tested for their reactivity's with isolates of the 22 serovars (see Methods). The cutoff values are depicted as horizontal bars and were defined as the mean plus 3 standard deviations obtained for sera from five healthy individuals. **A**, **B** and **C** show the data for Lsa66, Lp30 and rLIC12238, respectively.

To investigate whether LIC10258, LIC12880 and LIC12238 coding sequences are capable to promote an immune response from an infected host, we evaluated the reactivity of the proteins with antibodies present in serum samples of confirmed early (MAT -) and convalescent (MAT +) phases of leptospirosis patients. We performed an ELISA using 20 serum samples, 10 of each phase of the disease. The results depicted in fig. 9 show that although several samples presented IgM- and IgG-antibodies against the recombinant proteins in the convalescent phase, positive MAT serum samples, no or very low reactivity was achieved at the early phase of the disease where MAT was negative. The best performances were obtained with Lsa66 and rLIC12238 proteins (Fig. 9A and 9C). It is worth mentioning that 30% of ELISA IgG antibodies were detected with Lsa66 with MAT negative serum (Fig.9A).

## Discussion

A number of studies have established OmpA as a multifaceted molecule with many diverse roles. OmpA outer membrane protein of *Escherichia coli* and other enterobacteria have been reported to act as adhesin/invasin, to participate in biofilm formation and to have role as immune target and evasin [36]. OmpA-like domains (named after the C-terminal domain of *E. coli* OmpA protein) have been shown to non-covalently associate with peptidoglycan [37]. Members of this family include the *E. coli* outer membrane protein OmpA [36], the *E. coli* lipoprotein PAL (Peptidoglycan-associated lipoprotein) [38], the *Neisseria meningitidis* RmpM, a putative peptidoglycan binding protein, [39], the *E. coli* motor protein MotB [40] and the flagellar motor proteins PomB and MotY of *Vibrio alginolyticus* [41], which interact with the inner membrane. Moreover, peptidoglycan-associated lipoprotein (Pal) is a potential vaccine candidate against *Haemophilus influenza* [42]. The first protein identified in pathogenic *Leptospira* having a C-terminal OmpA consensus was Loa22 [43], followed by Omp52 identified in *L. santarosai* serovar Shermani, and shown to be reactive with sera of human patients infected with leptospires [44]. Recently, an OmpA70 was identified in *L. interogans* serovar Copenhageni [45]. The OmpA-like protein Loa22 was reported to be essential for the leptospiral virulence [43] and to promote inflammatory responses in cultured rat renal cells [46]. More recently, Yan and colleagues (2010) [47] have reported OmpA-like proteins as novel vaccine candidates for leptospirosis. Thus, it is possible that OmpA-like proteins have a function in leptospiral pathogenesis.

We have identified one novel putative protein with OmpA-like domain at C-teminus (OmpA C-like) encoded by the LIC10258 gene, we named Lsa66, and two putative lipoproteins, one encoded by the gene LIC12880, previously reported as Lp30 [17], and the other, LIC12238, described as a plasminogen-binding protein [15]. DNA amplification of LIC10258 was present in many serovars of *L. interrogans* but was absent in other pathogenic species tested, including *L. borgpertensenii* serovar Castellonis. Because this gene was identified in *L. borgpertensenii* serovar Hardjo-bovis genome sequences [32], it is possible that the gene is absent only in the serovar evaluated. Intriguingly, expression of LIC10258 was not observed in serovar Icteroharmorrhagiae under normal growth conditions. Likewise, no LIC12880 transcripts were detected in 3 pathogenic species of *Leptospira*. The gene LIC10258 was expressed only under physiological osmolarity in *L. interrogans* serovar Icterohamorrhagiae, a characteristic shared with *ligA* and *B* genes [48] and LIC10368 [8]. The addition of 120 mM NaCl to the culture medium reproduces the hosts serum osmolarity (∼300 mosM), thus providing a more physiological environment for leptospiral growth. Indeed, this is not surprising because 6% of the *L. interrogans* genes were revealed to be susceptible to osmoregulation [49]. Osmotic control of gene expression has been reported as an environmental cue associated with virulence in a variety of pathogens [34], including *Tcp pilli* of *V. cholerae* [50] and *invA* of *S. Typhimurium* [51]. Nevertheless, expression of the LIC12880 gene seemed to be restricted only to serovars of *L. interrogans* because bacterial cultures shifted to physiological conditions had no effect on gene expression.

The LIC10258, LIC12880 and LIC12238 coding sequences were cloned, without the signal peptide, and the proteins expressed in *E. coli* with a molecular mass of 66, 30 and 17 kDa, respectively. Recombinant proteins were purified by Ni^2+^-chelating chromatography as a major protein band and were recognized by anti-His tag monoclonal antibodies and by polyclonal antiserum obtained in mice against each of them. Assessment of secondary structure of the recombinant proteins after the purification process has been performed by CD spectroscopy and showed a mixture of α-helices and β-strands structures, similar to the data predicted by bioinformatics, rendering the recombinant proteins suitable for further studies.

The ability to interact with the host PLG system has been shown for several invasive gram-positive and gram-negative bacteria [52], [53]. Moreover, this interaction has been described for some virus and parasites [54], [55]. The plasminogen activation system was studied with several species of *Borrelia* and with *Treponema denticola* and suggested to have an important role during infection [56], [57]. We have reported that *Leptospira* species were also capable to bind PLG and generating plasmin, in the presence of activator, on the outer surface *in vitro* [14]. Furthermore, we have demonstrated that plasmin-coated virulent *L.interrogans* bacteria were capable of degrading purified extracellular matrix fibronectin, a step that may contribute to leptospiral invasiveness [14]. Verma et al. (2010) [58] have demonstrated that the protein LenA of *L. interrogans* [5], formerly LfhA/Lsa24 [6], [59], is a surface receptor for human plasminogen. More recently, we have shown eight novel PLG-receptor proteins of *Leptospira*, including rLIC12238 of this work [15]. We now identified Lsa66 and Lp30, as novel PLG-binding proteins. The highest binding affinity was achieved for rLIC12238 (*K*_D_ = 11.97±1.06 nM, in [15]) followed by Lsa66 (*K*_D_ = 68.82±25.24 nM), being the lowest value for Lp30 (*K*_D_ = 167.39±60.14 nM). The binding affinity value obtained for Lsa66 is of the same order of magnitude of the ones calculated for other recombinant proteins reported from our laboratory [15]. Bound PLG, as demonstrated with rLIC12238, could be converted to plasmin by the addition of urokinase-type PLG activator (uPA), showing specific proteolytic activity. Thus, it is possible that this protein may contribute to leptospiral infectiveness.

Several leptospiral adhesins have been described to date**.** These include 36-kDa fibronectin-binding protein [60] **,** Lsa24 [6] /LfhA [59], LigA and LigB proteins [7], Len-family proteins [5], Lsa21 [8], LipL32 [9], Lsa27 [61], Lp95 [11], TlyC [10], LipL53 [30], Lsa63 [12] and OmpL37 [13]. Lsa66 exhibits extracellular matrix-binding properties, and it is a laminin and plasma fibronectin-binding protein. Estimated binding affinity of Lsa66 was higher to laminin than with plasma fibronectin. It is thus possible that besides acting as PLG-receptor, this protein may also play a role in the attachment to host tissues.

Lsa66 inhibited adhesion of intact *L. interrogans* to immobilized plasminogen, laminin and plasma fibronectin. Comparable effect was exhibited by Lp30 upon plasminogen. The inhibitory effect promoted by the recombinant proteins was partial and in agreement with the presence of additional binding proteins contributing to the leptospiral adherence to extracellular matrix components and plasminogen. Similar results were obtained with other leptospiral adhesins, Lsa24 [6], LigA and LigB proteins [7], Lsa63 [12] and OmpL37 [13].

Immunofluorescence of LIC10258, LIC12880 and LIC12238 coding sequences at the surface of leptospires, as a result of antiserum recognition raised against each protein was detected as single spots. This could be explained since quantitative proteomics studies of *L. interrogans* have estimated only 75 and 11 copies per cell of LIC10258 and LIC12238, respectively, while LIC12880 did not fall in protein abundance scale to have its number of copies calculated [62]. On the other hand, 3,800 and 15,000 copies per cell were estimated of LipL32 and GroEL, respectively. These findings could explain the focal distribution and low fluorescence intensity of our coding sequences, contrasting with the fluorescence detected for LipL32. Interestingly, although present in high copy number, no green fluorescence was detected when anti-GroEL was added, attesting the integrity of the bacteria.

Lsa66 and rLIC12238 share serum recognition properties with the previously described adhesins, LipL32 [63], LigA and LigB proteins [64], Lsa27 [61], Lsa63 [12] and OmpL37 [13] that have shown positive reactivity with serum samples from patients diagnosed with leptospirosis. Interestingly, the OmpA-like leptospiral lipoprotein Loa22, although reactive with convalescent mouse sera and essential for virulence of *L. interrogans* in the animal model [43], does not bind ECM molecules [6]. This suggests that the binding of Lsa66 to laminin or plasma fibronectin does not occur through the OmpA-like domain at the C-terminus. Protein recognition by serum of confirmed leptospirosis samples together with the immunofluorescence data suggest that Lsa66 and rLIC12238 may be surface exposed.

In conclusion, we present in these studies one novel OmpA-like protein, Lsa66, and two predicted lipoproteins that are PLG-binding receptors. Lsa66, in addition, is an ECM binding protein that reacts with antibodies present in both phases of the disease. Thus, it is possible that this dual activity of Lsa66 may promote the attachment to host via ECM and may help the leptospires to overcome tissue barriers by plasmin generation. Revealing bacteria–host interactions at a molecular level should elucidate our understanding of the host physiology and facilitate the search for vaccine targets against leptospirosis.
